# Diseases of the Skin

**Published:** 1886-01

**Authors:** 


EPITOME OF DISEASES OF THE SKIN, BEING AN ABSTRACT OF A COURSE OF LECTURES DELIVERED IN THE UNIVERSITY OF PENNSYLVANIA, DURING THE SESSION OF 1883–4. *By* LOUIS A. DUHRING, M. D., *Professor of Skin-Diseases. Reported by* HENRY WILE, M. D., *Clinical Assistant in the Department of Skin Diseases in the University Hospital; Philadelphia*: J. B. LIPPINCOTT COMPANY, *Chicago*, 1885, PP. 130.

This miniature treatise on cutaneous diseases will commend itself to the profession, rather by the just eminence of the author's name, than by its intrinsic excellence. Few beside students of medicine are capable of appreciating in the highest degree a work so small that it dismisses a disease in a paragraph. Still, if adversity has its uses, so also have the helps, the hand-books, the epitomes and the manuals. Among them there are none so useful as those where the abstract is made, not by a penny-a-liner, but by the master himself. Among all the epitomes, therefore, judged by this fair standard, there is none better than Dr. During's useful little compendium.

---

THE TECHNOLOGY OF BACTERIA INVESTIGATION. *Explicit directions for the study of Bacteria, their culture, staining, mounting, etc, according to the methods employed by the most eminent investigators.* By CHARLES S. DOLLEY, M.D., *Boston:* S. S. CASSINO & CO, 1885. *Chicago:* W. T. KEENER.

It could hardly be expected that DR. DOLLEY, in so small a work, would exhaust the subject he has endeavoured to treat. In his preface he states that his work is put forth with the view of stimulating careful study of schizomycetes by American investigators.

He has conveniently grouped his material under three heads—I. General Directions. II. Special Methods for Investigating Pathogenic Bacteria. III. Formulary. Under part I. he reviews the most approved methods of obtaining and studying micro-organisms, (a) in the living state; (b) fixed, hardened and stained, including an allusion to the photographic branch of bacterial study. The author treats very exhaustively of the different modes of cultivation; devoting, however, surprisingly little space to vaccination and inoculation experiments. Part I. closes with some remarks on what the writer styles "Biological Analysis", in which there is nothing new or especially interesting.

In part II. the different recognized specific micro-organisms in disease are described, but we regret that the material collected on such subjects as malaria, diphtheria, syphilis and yellow-fever is so meagre. The specific poisons of scarlatina, measles, meningitis, etc., are merely alluded to.

In part III. viz. *Formulary*, the directions for the preparation of staining fluid, culture media, instructions as regards mounting specimens, etc., are very full and clear, making the book a valuable one for the laboratory.

The work is placed before the public in an excellent form, but we regret that it is devoid of illustrations, for in no subject we believe, are illustrations more necessary. We believe it to be a book which the profession needs, and which will fully repay any one who will read what the author has so carefully collected and systematically grouped. At the end of each section, references are given to the different authorities making it very easy to go back to the original sources of information.

F.C.

### BOOKS RECEIVED.

BOOKS AND PAMPHLETS RECEIVED.

*Fownes's Manual of Chemistry*. Philadelphia: Lea Brothers & Co. Chicago: Jansen, McClurg & Co.

*A System of Obstetric Medicine and Surgery*. By Robert Barnes, M. D., and Fancourt Barnes, M. D. Philadelphia: Lea Brothers & Co. Chicago: Jansen, McClurg & Co.

*A Text-Book of Pharmacology, Therapeutics and Materia Medica*. By T. Lauder Brunton, M. D., D. SC., F. R. S. Philadelphia: Lea Brothers & Co. Chicago: Jansen, McClurg & Co.

*The Essentials of Histology*. By E. A. Schäfer, F. R. S. Philadelphia: Lea Brothers & Co. Chicago: Jansen, McClurg & Co.

*A System of Practical Medicine by American Authors*. Edited by William Pepper, M. D., LL. D. Philadelphia: Lea Brothers & Co. Chicago: Jansen, McClurg & Co.

*A Practical Treatise on the Diseases of Children*. By Alfred Vogel, M. D. New York: D. Appleton & Co. Chicago: Jansen, McClurg & Co.

*The Science and Art of Midwifery*. By William Thompson Lusk, A. M., M. D. New York: D. Appleton & Co. Chicago: Jansen, McClurg & Co.

*Manual of the Diseases of Women*. By Charles H. May, M. D. Philadelphia: Lea Brothers & Co. Chicago: Jansen, McClurg & Co.

*A Text-Book of Nursing*. Compiled by Clara S. Weeks. New York: D. Appleton & Co. Chicago: Jansen, McClurg & Co.

*Rationalism in Medical Treatment, or the Restoration of Chemism*. By William Thornton. Boston: Published by the author.

*Clinical Therapeutics*. By Prof. Dujardin-Beaumetz. Detroit: George S. Davis.

*Post-Mortem Examinations: with Especial Reference to Medico-Legal Practice*. By Professor Rudolph Virchow. Philadelphia: P. Blakiston, Son & Co. Chicago: W. T. Keener.

*Epilepsy and Other Chronic Convulsive Diseases*. By W. R. Gowers, M. D., F. R. C. P. New York: William Wood & Co. Chicago: W. T. Keener.

*A Reference Hand-Book of the Medical Sciences*. Edited by Albert Buck, M. D. New York: William Wood & Co. Chicago: W. T. Keener.

*Epitome of Diseases of the Skin*. By Louis A. Duhring, M. D. Philadelphia: J. B. Lippincott & Co. Chicago: W. T. Keener.

*Practical Surgery*. By J. Ewing Mears, M. D. Philadelphia: P. Blakiston, Son & Co. Chicago: W. T. Keener.

*Applied Medical Chemistry.* By Lawrence Wolff M. D. Philadelphia: P. Blakiston, Son & Co. Chicago: W. T. Keener.

*Milk-Analysis and Infant-Feeding.* By Arthur V. Meigs, M. D. Philadelphia: P. Blakiston, Son & Co. Chicago: W. T. Keener.

*Transactions of the Texas State Medical Association.* 1885.

*The Physician's Visiting-List for 1886.* Philadelphia: P. Blakiston, Son & Co. Chicago: W. T. Keener.

*Acne, its Etiology, Pathology and Treatment,* by L. Duncan Bulkley, A. M., M. D. New York: G. P. Putnam's Sons.

*Practical Therapeutics:*—A compendium of selected formulas and practical hints on treatment, by Edward J. Bermingham, A. M., M. D. New York: J. R. Bermingham.

*A Treatise on Nervous Diseases;* their Symptoms and Treatment, by Samuel G. Webber, M. D. New York: D. Appleton & Co. Chicago: Jansen, McClurg & Co.

*The Use of the Microscope in Clinical and Pathological Examinations,* by Dr. Carl Friedlander. New York: D. Appleton & Co. Chicago: Jansen, McClurg & Co.

*The Management of Labor and of the Lying-In Period,* by Henry G. Landis, A. M., M. D. Philadelphia: Lea Bros & Co. Chicago: Jansen, McClurg & Co.

*Applied Medical Chemistry,* by Lawrence Wolff, M. D. Philadelphia: P. Blakiston Son & Co. Chicago: W. T. Keener.

*A Text-Book of Medical Chemistry,* by Elias H. Bartley, M. D. Philadelphia: P. Blakiston Son & Co. Chicago: W. T. Keener.

*Tracts on Massage No. 11; the Physiological Effects of Massage,* by Benjamin Lee, A. M., M. D., Ph. D. Philadelphia. 1885.

*The Pedigree of Disease,* by Jonathan Hutchison, F. R. S. New York: William Wood & Co. Chicago: W. T. Keener.

*Inorganic Chemistry,* by Edward Frankland and Francis R. Japp. Philadelphia: Lea Brothers & Co. Chicago: Jansen, McClurg & Co.

*Cutaneous Memoranda,* by Henry G. Piffard, A. M., M. D. Third Edition. New York: William Wood & Co. Chicago: W. T. Keener.

*Venereal Memoranda,* by P. A. Morrow, A. M., M. D. New York: William Wood & Co. Chicago: W. T. Keener.

*Facts and Mysteries of Spiritism,* by Joseph Hartman. Philadelphia: Thomas W. Hartley & Co. Chicago: Jansen, McClurg & Co.

*Text-Book of Ophthalmoscopy,* by Edward G. Loing, M. D. New York: D. Appleton & Co. Chicago: Jansen, McClurg & Co.

*Climatology and Mineral Waters of the United States,* by A. N. Bell, A. M., M. D. New York: William Wood & Co. Chicago, W. T. Keener.

*Diseases of the Lungs,* by Prof. Germain Sée. Translated by E. P. Hurd, M. D. Chicago: W. T. Keener. New York: William Wood & Co.

*Diagnosis of Diseases of the Brain and of the Spinal Cord,* by W. R. Gowers, M. D., F. R. C. P. New York: William Wood & Co. Chicago: W. T. Keener.

### PAMPHLETS RECEIVED.

*Surgical Treatment of Cysts of the Pancreas.* By N. Senn, M. D.

*Iritis: Its Relation to the Rheumatic Diathesis and its Treatment.* By Charles J. Lundy, A. M., M. D.

*The Induction of Premature Labor in Certain Cases.* By Walter Coles, M. D.

*Note on a Form of Post-Neuralgic Insanity.* By C. H. Hughes, M. D.

*The Role of Bacteria in Parturition.* By Henry O. Marcy, A. M., M. D.

*A Case of Membranous Occlusion of the Posterior Nares*, by W. E. Casselberry, M. D.

*A New Bandage for Fixation of the Humerus and Shoulder-Girdle*, by Charles W. Dulles, M. D.

*The External Therapeutics of Pulmonary Consumption*, by Thomas J. Mayo, M. D.

*Some of the Surgical Sequelæ of the Exanthenes and Continued Fevers*, by Roswell Park, A. M., M. D.

*Address of the State Board of Health to the People of Pennsylvania.*

*Observations on the Cause and Treatment of Infantile Eczema and Allied Eruptions*, by Henry T. Byford, M. D.

*Abnormal Positions of the Head. What do they Indicate?* By Edward Borck, A. M., M. D.

*The Respiratory Function of the Human Larynx*, by Franklin H. Cooper, M. D.

*A New Electric Light.* Dr. William Chapman Jarvis.

*Clinical Notes on Uterine Surgery.* J. Marion Sims, A. B., M. D. New York: William Wood & Co. Chicago: W. T. Keener.

*Effects of the Use of Tobacco.* Hobart Amory Hare, M. D.

*Report of the Committee on Disinfectants of the American Public Health Association.*

*Transactions of the American Ophthalmological Society.*

*Hereditary or Degenerative Ataxia.* W. Everett Smith, M. D.

*Aids to Surgery.* George Brown, M. R. C. S., L. S. A.

*Aids to Obstetrics.* Samuel Hall, B. A., M. D.

*Aids to Medicine.* C. E. Armand Semple, B. A., M. D,

*Aids to Gynæcology.* Alfred S. Guebb, L. R. C. P., M. R. C. S.

*Manuel des Injections sous-cutanees. Par Bourneville et Bricon.*

*Observations upon the Mutual Relations of the Medical Profession and the State.* Donald Maclean, M. D.

*Treatment of the Umbilicus in the New Born.* E. H. King, M. D.

*Transactions of the American Otological Society.*

